# A Review on Agro-Waste-Derived Carbon Dots as Multifunctional Nanofillers in Biopolymer Films: A Sustainable Strategy for Active Packaging and Shelf Life Extension of Seafood

**DOI:** 10.3390/foods15091594

**Published:** 2026-05-04

**Authors:** Arunachalasivamani Ponnusamy, Yadong Zhao, Bin Zhang, Soottawat Benjakul

**Affiliations:** 1 International Center of Excellence in Seafood Science and Innovation, Faculty of Agro-Industry, Prince of Songkla University, Hat Yai 90110, Songkhla, Thailand; 6511030004@email.psu.ac.th; 2 Key Laboratory of Health Risk Factors for Seafood of Zhejiang Province, College of Food Science and Pharmacy, Zhejiang Ocean University, Zhoushan 316022, China; yadong@kth.se (Y.Z.); zhangbin_ouc@163.com (B.Z.); 3 BioNanocomposite Research Center, Department of Food and Nutrition, Kyung Hee University, 26 Kyungheedae-ro, Dongdaemun-gu, Seoul 02447, Republic of Korea

**Keywords:** carbon dots, agro-waste valorization, active packaging, seafood preservation, biopolymer nanocomposites, shelf life extension

## Abstract

Seafood, rich in nutrients, undergoes rapid quality deterioration, primarily due to microbial activity and lipid oxidation. Conventional petroleum-based packaging is widely used for seafood but lacks the ability to retard spoilage. Carbon dots (CDs), which are nanosized, act as multifunctional additives that can be incorporated into biopolymer films to prepare active, biodegradable packaging. CDs are produced through green synthesis methods using various agro-byproducts, including fruit peels, leaves, and rhizomes, thus aligning well with circular economy principles. CDs have antioxidant and antimicrobial activities, as well as UV barrier properties. CDs from different sources show varying bioactivities and properties. The bioactivities of CDs are enhanced by doping with compounds such as polyphenols and amino acids. When CDs are applied to biopolymer matrices such as chitosan and gelatin, the increases in mechanical strength, water vapor barrier properties, thermal stability, and ultraviolet light-blocking ability can be achieved. Recent investigations into the performance of films containing CDs from different sources for the shelf life extension of various seafood are revisited. The limited commercial implementation, particularly of large-scale synthesis, is addressed. The migration behavior and toxicological profiles are also elucidated. Overall, this review highlights agro-waste-derived CDs as a potential nanomaterial for developing next-generation active packaging systems for seafood preservation and environmental sustainability.

## 1. Introduction

Seafood is a staple component of the human diet in many regions and is valued for its high nutritional benefits [1]. It is particularly rich in proteins (15–25%) and possesses several essential amino acids, including histidine, isoleucine, leucine, lysine, methionine, phenylalanine, threonine, tryptophan, and valine [2]. Seafood is also an important source of Ω-3 fatty acids [1], a range of water- and fat-soluble vitamins, and minerals such as iodine, selenium, calcium, and phosphorus, etc. Fresh seafood is extremely vulnerable to microbial and oxidative deterioration during processing, distribution and storage. This deterioration leads to quality loss, limited shelf life, and reduced consumer safety, and contributes to substantial food loss. Conventional packaging materials, predominantly petroleum-based plastics, are used to protect food from environmental stress and also delay quality deterioration to some extent during storage and distribution. These packaging materials are made from non-biodegradable plastics, leading to detrimental environmental impacts after use.

Biodegradable packaging made from proteins, polysaccharides, lipids, or their combination provides an eco-friendly, Bio-Circular-Green-based sustainable solution to plastic pollution. Frequently utilized biopolymers for seafood packaging include chitosan (CS), gelatin (GL), starch (ST), alginate, and cellulose derivatives. These materials are biodegradable, renewable, and generally recognized as safe (GRAS) [3]. However, they exhibit inadequate mechanical strength, insufficient water vapor barrier properties, and constrained functional performance. Moreover, the conventional packaging materials do not effectively inhibit spoilage of packed foods during the extended storage [4], thereby creating an urgent need for innovative packaging strategies. One strategy is active packaging, in which active fillers with antioxidant and/or antimicrobial properties are incorporated into biopolymer films. These films provide the advantage of effective food preservation with extended shelf life while minimizing environmental pollution.

Active packaging is engineered to deliberately incorporate active components that can eradicate spoilage bacteria and pathogens in contaminated food or from the environment. Consequently, seafood can have a prolonged shelf life and its safety can be enhanced, hence meeting consumer demand for fresh and safe foods [5]. Recently, carbon dots (CDs), zero-dimensional carbon-based nanoparticles, have attracted significant attention due to their unique attributes for active food preservation [6]. CDs exhibit quantum confinement effects and distinctive physicochemical properties, including high fluorescence, excellent water solubility, high stability, and minimal toxicity [7]. These CDs can be synthesized by various carbonization methods involving high temperatures, pressure, sonication, etc. Among them, hydrothermal synthesis is a green approach due to its relatively mild conditions, simplicity, and cost-effectiveness [8]. Significantly, agricultural wastes or byproducts, such as fruit peels [7], leaves [9], rhizomes [10], and seafood waste, serve as abundant, low-cost precursors for the synthesis of CDs. Their production closely aligns with circular economy principles (CEPs) by maximizing the value of underutilized biomass or residues via conversion into high-value functional nanomaterials or products.

Relevant to food preservation, CDs offer a unique combination of antioxidant and antimicrobial properties with good biocompatibility [11]. The antioxidant capacity of CDs arises from functional groups on the carbonic core that can scavenge free radicals and reactive oxygen species (ROS) [12]. The antimicrobial activity depends on the particle size and surface charge of the CDs, which effectively suppress common seafood spoilage and/or pathogenic bacteria, including *Pseudomonas* spp., *Listeria* spp., and others. CDs also exhibit strong ultraviolet (UV) light-blocking capacity, protecting light-sensitive nutrients from photodegradation [13]. CDs derived from different precursors display diverse bioactivities and physicochemical characteristics [14]. Furthermore, the bioactivities of CDs can be tailored or enhanced by doping with other functional compounds, such as polyphenols [15], amino acids and organic acids [14].

In recent decades, CDs have been documented to enhance mechanical integrity. They also provide higher thermal stability, superior UV-blocking properties, and controlled release of active compounds into the food matrix [7]. The efficacy of CDs in prolonging the shelf life of various seafood products, particularly when incorporated into CS films, has been documented [7,16,17]. CDs have been documented to prolong the shelf life of chilled fish [17], shrimp [16], clams [18], and other species by inhibiting microbial proliferation and reducing oxidative damage. These CD-based films have also preserved quality attributes, including color, texture, and sensory characteristics, of seafood throughout refrigerated storage [5]. Despite these promising impacts, numerous challenges and limitations still impede their widespread commercial use.

## 2. Seafood Quality Deterioration, Spoilage Mechanisms and Challenges

**Figure 1:**
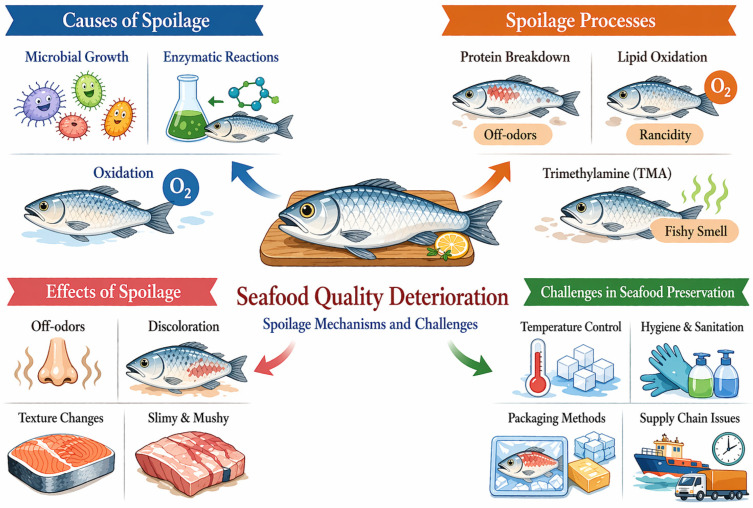
Overview of seafood quality deterioration, spoilage mechanisms, and associated challenges.

Seafood, comprising fish, shrimp, crabs, and mollusks, exhibits unique nutritional properties in comparison to terrestrial animal products [1]. It is abundant in high-quality proteins that contain all necessary amino acids in adequate ratios, closely aligning with human nutritional needs. Due to the activity of specific spoilage organisms (SSOs), decomposition products and diverse metabolites are accumulated, resulting in off-odors (Figure 1). The composition and activity of SSOs fluctuate with harvest environment, handling procedures, processing conditions, storage temperature, and packaging systems [19]. *Pseudomonas* spp. represent the dominant spoilage organisms for aerobically stored fresh seafood. These Gram-negative bacteria generate volatile compounds responsible for unpleasant odors. *Shewanella* spp. are particularly important in refrigerated seafood spoilage as they reduce trimethylamine N-oxide to trimethylamine (TMA), producing characteristic fishy odors. *Photobacterium phosphoreum*, a psychrotolerant bacterium, dominates during the spoilage of seafood stored under modified atmosphere packaging. Lactic acid bacteria dominate in vacuum-packaged seafood. Enterobacteriaceae contribute to spoilage under poor hygienic practices [20].

On the other hand, lipid oxidation in fish muscle occurs through intricate chemical pathways. Oxidation begins with the formation of free radicals, typically triggered by light, heat, myoglobin, or metal ions [21]. Highly unsaturated fatty acids easily lose hydrogen atoms, producing lipid radicals that interact with molecular oxygen to create peroxyl radicals and hydroperoxides. The propagation of these chain reactions results in the continuous generation of free radicals. Hydroperoxides, the principal oxidation products, are often devoid of taste and odor [22]. However, they undergo decomposition into secondary products, such as aldehydes, ketones, alcohols, hydrocarbons, and volatile organic compounds, through cleavage, rearrangement, and further reactions [21]. These secondary oxidation products are responsible for rancid aroma and undesirable fishy flavor that can be perceived at minimal concentrations. Hexanal, propanal, and malondialdehyde serve as the prevalent markers of lipid oxidation, which mainly contribute to off-odors in the oxidized seafood [23].

Microbial spoilage and lipid oxidation do not occur independently. These reactions progress synergistically to expedite the product’s overall quality deterioration. Certain microbes may adapt and metabolize the oxidation products, especially aldehydes, as carbon sources. For example, hexanal and analogous aldehydes can be employed by *Pseudomonas* spp. through enzymatic oxidation to yield the corresponding carboxylic (COO^−^) acids [24]. This adaptation supports bacterial proliferation as the oxidation process advances, effectively linking microbial and oxidative spoilage pathways. In addition, bacterial lipases hydrolyze triglycerides, releasing free fatty acids that are more prone to oxidation. Microbial metabolism generates characteristic amines, sulfides, and organic acids, while oxidative rancidity yields aldehydes, ketones, and alcohols [21]. As seafood quality declines, both consumers and quality assessors rely heavily on changes in odor. The interplay between microbial and oxidative volatiles, however, produces complex sensory profiles, with some microbial metabolites exerting pro-oxidant or antioxidant effects depending on their structure.

Protein oxidation often accompanies lipid oxidation. ROS and lipid-derived oxidation products attack susceptible amino acid side chains, particularly those containing sulphur (methionine, cysteine) and aromatic rings (tyrosine, phenylalanine), leading to alterations in texture and water-holding capacity [21]. The resulting moisture loss and structural degradation further promote microbial growth by releasing more accessible nutrients for SSOs proliferation [20]. Collectively, these interconnected processes complicate shelf life prediction when only a single quality index is used.

During storage, seafood undergoes progressive physical, chemical, and sensory changes. Texture deteriorates as endogenous and microbial proteolytic enzymes degrade muscle and collagenous proteins, resulting in softening, gaping, and loss of firmness [25]. Color changes occur due to pigment oxidation and non-enzymatic reactions; the initial fresh, translucent appearance gradually becomes opaque and discolored. Flavor deterioration involves multiple concurrent mechanisms. The characteristic fresh flavor, derived from nucleotides, free amino acids, and small carbohydrates, diminishes rapidly. Bitter compounds can be generated and accumulated via proteolysis and nucleotide degradation, while rancid flavors emerge from lipid oxidation products. Increasingly unpleasant flavors from microbial metabolism further contribute to quality loss. Odor changes, in particular, provide the most recognizable spoilage indicator [26]. Fresh seafood aromas, often described as sea-like, sweet, or cucumber-like for certain species, fade during spoilage, whereas neutral or stale odors and eventually offensive putrid smells become dominant, particularly mediated by ammonia, sulphur compounds, and biogenic amines [27].

Nutritional quality also declines during storage. Levels of polyunsaturated fatty acids (PUFA) tend to decrease due to oxidation, and essential amino acids become less bioavailable. Vitamins, especially fat-soluble forms, are degraded by oxidative processes [1]. These nutritional losses diminish the health benefits typically associated with seafood consumption.

Substantial biological variability across species, seasons, and harvest locations leads to inconsistent raw material quality. Fishing and post-harvest handling, particularly temperature control, are critical for preserving quality. Different categories of seafood, such as fatty fish, lean fish, and shellfish, exhibit distinct spoilage susceptibilities and thus require properly tailored preservation strategies [28]. At the same time, consumer preferences for fresh, minimally processed products with limited use of synthetic preservatives restrict the applicability of some conventional preservation methods, whereas the demand for innovative technologies has been increasing.

Logistical constraints in distribution, including maintaining an uninterrupted cold chain and long transport distances, further increase the effectiveness of the technology, making it more advantageous than traditional packaging. Conventional petroleum-based packaging materials offer only passive protection. They act as inert physical barriers without directly inhibiting microbial growth or oxidative reactions in packaged seafood [4]. They generally lack oxygen- and radical-scavenging activity (RSA), offer limited light protection, and lack intrinsic antimicrobial activity. Simultaneously, environmental concerns exacerbate the global problem, as these plastics persist for centuries. These limitations justify the development of advanced active biodegradable packaging systems. Biopolymer films reinforced with CDs derived from agro-waste hold the promise for enhancing seafood preservation efficacy and reducing environmental impact.

## 3. Valorization of Agro-Waste: A Circular Economy Approach

Agro-industrial and agricultural activities generate large quantities of waste materials, including fruit peels, seeds, pomace, shells, husks, straw, and bagasse, etc. The accumulation of these residues poses significant environmental and economic challenges worldwide [29]. Conventional waste management strategies, such as open dumping and landfilling, require substantial land area and can release methane and toxic substances. Open burning yields particulate matter and other pollutants, which can contaminate the atmosphere [30]. These practices also represent the missed opportunities for resource recovery and better utilization. The environmental burden extends beyond disposal sites, as wasted food components embody large inputs of land, water, energy, and agrochemicals [31]. Consequently, reducing waste generation and recovering valuable substances from byproducts can contribute to improved sustainability.

CEPs offer an alternative to the traditional disposal model by seeking to keep resources in use as much as possible through waste reduction, reuse, recycling, and energy recovery. When applied to agro-industrial waste streams, CEPs reveal substantial opportunities because these byproducts contain valuable constituents, including carbohydrates, proteins, fibers, pigments, and bioactive phytochemicals [32]. Such components can serve as feedstocks for new materials and products. This strategy not only mitigates waste disposal challenges but also creates economic value from materials, a waste-to-wealth approach—previously regarded as low-value or burdensome. The biological cycle is particularly relevant for agro-waste, as organic residues can ultimately return safely to the biosphere via composting or anaerobic digestion [31]. However, higher-value products can be obtained through material recovery prior to biological degradation. This can optimally maximize the efficiency of resource use and exemplifies cascading use, a core principle of CEPs.

**Figure 2:**
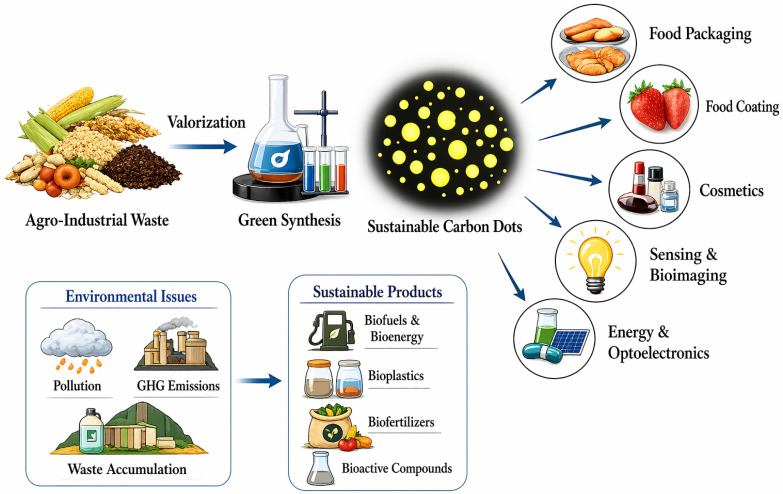
Schematic illustration of agro-industrial waste valorization into carbon dots following a circular economy approach.

Agro-wastes generated from industries that utilize agricultural productsthat are processed into food or other products, e.g., food processing industries, etc., hold substantial potential as precursors for CDs synthesis (Figure 2). These residues contain abundant carbon, hydrogen, oxygen, and nitrogen derived from lignocellulosic components, proteins, and polyphenols [33]. Wastes from fruit and vegetable processing are especially attractive, including citrus peels (orange, lemon, grapefruit), which are rich in flavonoids, and banana peels, which are high in potassium. Peels from apple pomace and pineapple contain residual sugars, polyphenols, essential oils, pectin, and cellulose. Furthermore, mango stones, pomegranate rinds, and watermelon rinds have all been successfully converted into CDs [34]. Root, tuber, and legume processing wastes, such as cassava peels, sweet potato residues, yam peels, soybean hulls, and okara, can serve as potential substrates that can facilitate in situ heteroatom doping during CDs formation [35].

Table 1 summarizes CDs derived from agro-waste involving precursors, synthesis methods, conditions, properties, and applications. CDs derived from agro-waste offer multiple benefits. Environmentally, they transform problematic residues into value-added products while reducing the burden on waste management or treatment systems [33]. Economically, the raw material costs are low or even negative, since many agro-wastes incur high costs for disposal. CDs production thus presents opportunities for rural economic development through decentralized processing facilities near waste generation sites. Functionally, the complex composition of agro-wastes, including naturally occurring heteroatoms and phytochemicals, supports in situ doping and the formation of functional surface groups, enabling property tuning via precursor selection [36]. From a processing standpoint, green synthesis routes typically use water as the solvent and operate under relatively mild conditions, avoiding hazardous organic solvents and simplifying scale-up [37]. Collectively, these environmental, economic, functional, and practical advantages support sustainable CDs manufacturing aligned with CEPs.

Despite these advantages, several challenges must be addressed. The composition of agro-waste varies with season, geographical origin, and storage conditions, complicating standardization and quality control. Contaminants such as pesticide residues, heavy metals, and microbial loads may persist through processing, thus requiring rigorous source verification and testing protocols for food-contact applications [49]. Collection and storage logistics are also complex because waste is often geographically dispersed, seasonally available, and susceptible to deterioration during storage, all of which affect commercial feasibility. Scaling up green synthesis methods demands the optimization of continuous hydrothermal reactors, energy efficiency assessments, and purification strategies suitable for high-throughput production [6]. Furthermore, agro-waste often has competing uses, such as animal feed, compost, and bioenergy production. Nonetheless, CDs production must demonstrate clear economic and sustainability advantages over feedstock allocation.

CDs production from agro-waste embodies the CEPs by reintegrating waste materials into productive use rather than requiring new resources. This approach advances several Sustainable Development Goals (SDGs), including responsible consumption and production (SDG 12) through waste valorization [36], climate action (SDG 13) by reducing methane emissions, and life below water (SDG 14) via decreased plastic pollution when CDs are embedded in biodegradable packaging materials. The cascading use concept can be applied throughout the value chain. After CDs synthesis, the remaining solid residues from hydrothermal processing may serve as soil amendments or bioenergy feedstocks, thereby maximizing resource utilization before final return to the biosphere [50]. Local or regional production schemes, in which agricultural areas convert their own waste into CDs for nearby packaging applications, align with distributed manufacturing models.

## 4. Carbon Dots: Synthesis, Characteristics, Bioactivities and Cytotoxicity

### 4.1. Green Synthesis Methodologies and Agro-Waste-Derived Carbon Dots

**Figure 3:**
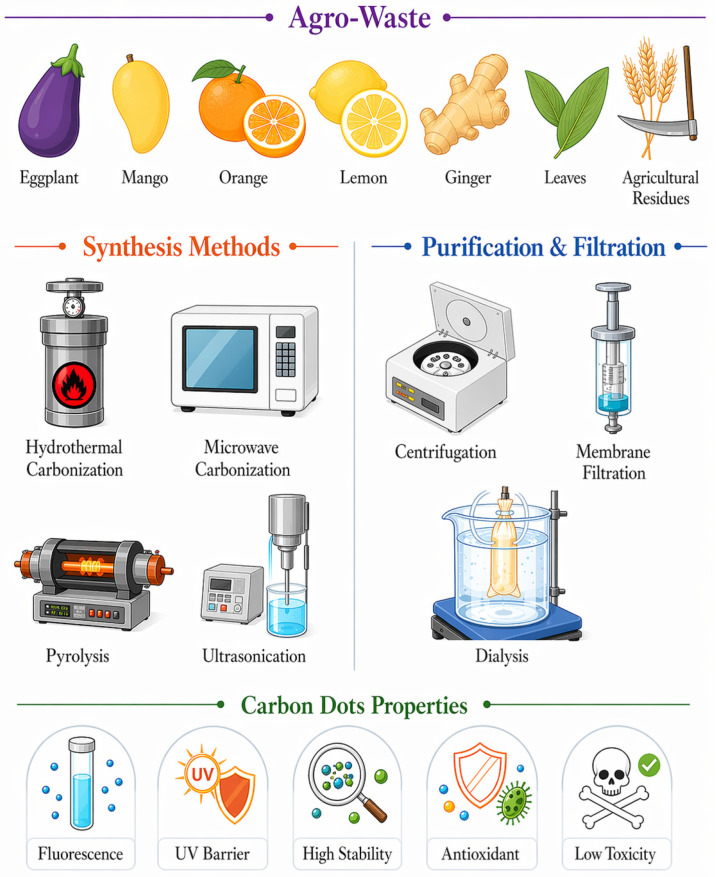
Schematic overview of carbon dots derived from agro-waste: synthesis methods, physicochemical characteristics, and bioactivities.

The production of CDs from agro-wastes is consistent with green chemistry concepts by reducing environmental impact and enhancing value compared to conventional synthesis routes that rely on harsh solvents. Various green approaches have been developed. Each method shows distinct advantages and disadvantages based on the type of waste and the desired CDs functionalities (Figure 3).

Hydrothermal carbonization is the predominant synthesis method. It employs water as the reaction medium in enclosed Teflon reactors, and the reaction is generally carried out at 150–250 °C for several hours [36]. The process is comparatively simple and produces CDs with numerous oxygen-containing surface groups [51]. Microwave-assisted synthesis provides a rapid and energy-efficient alternative, with reaction times decreasing from hours to minutes. The rapid volumetric heating produced by microwaves can generate smaller, more uniform particles with lower overall energy consumption. Thus, this method is suitable for laboratory-scale and continuous processes [36].

Pyrolysis occurs under inert atmospheres at temperatures ranging from 300 to 800 °C. This method is effective for high-lignin-containing wastes such as nutshells and husks [12]. However, the increased temperatures associated with high pressure and potential generation of undesirable byproducts require meticulous regulation, centrifugation, and purification. Furthermore, optimization is required for the production of CDs from different precursors. Solvothermal methods require eco-friendly green solvents, including water, ethanol, and deep eutectic solvents, which offer additional options for tailoring CDs properties [52]. These solvents are GRAS, biodegradable, and recoverable, thereby providing tunable physicochemical environments for specialized CDs synthesis [53].

Ultrasonic-assisted synthesis employs high-frequency sound waves to enhance carbonization and fragmentation. The collapse of cavitation bubbles generates localized hotspots with elevated temperature and pressure, thus promoting bond cleavage and CDs formation at lower temperatures [54]. Ultrasound can be integrated with other carbonization methods, including hydrothermal or microwave techniques, to achieve the enhanced performance as reflected by the improved yield, fluorescence and bioactivities [36].

The choice of method is contingent upon several factors, including precursor type, desired CDs size, charge, surface chemistry, and intended production scale [55]. The transformation of agricultural biomass into versatile CDs commences with the dehydration and depolymerization of primary constituents. During carbonization, polysaccharides undergo cleavage at glycosidic bonds, proteins are hydrolyzed, and lignin is decomposed into smaller molecules [51]. With a further increase in temperature, the intermediates such as hydroxy-methyl furfural are formed and undergo polymerization, cyclization, and condensation to form new aromatic structures [56]. Subsequently, carbonization occurs via nucleation and development of small aromatic clusters that coalesce into particles featuring carbonaceous cores with functionalized surfaces. Surface functionalization occurs concurrently, with oxygenated/nitrogen-containing groups incorporated into the carbonic framework of CDs [51]. These active groups on the surface of CDs govern the solubility, reactivity, and interactions of CDs with polymers or other substances. The final product generally comprises heterogeneous carbon nanoparticles with reactive functional groups on the surface of graphitic or amorphous cores [56]. To obtain pure CDs, purification steps such as centrifugation, filtration, and dialysis are required. The particle size distribution can be controlled by removing residual precursors and byproducts. After purification, the sizes of the formed CDs range from 2 to 10 nm, where quantum confinement effects elicit fluorescence at different excitation and emission wavelengths [14]. CDs sourced from a variety of agro-wastes, such as fruit-processing residues, vegetable wastes, cereal and grain byproducts, beverage-industry byproducts, rhizomes, etc., exhibit varying properties and bioactivities [7,10,57].

### 4.2. Factors Influencing Physicochemical Properties of Carbon Dots

The composition of the precursor fundamentally determines the CDs structure and functionalities. The overall carbon content of the source material affects core formation, while nitrogen and sulphur constituents enable heteroatom doping, which modifies the electronic structure and bioactivity, particularly at the surface of CDs [14]. The reaction temperature is a critical parameter, with lower temperatures (<200 °C) tending to produce CDs with more oxygenated groups and predominantly amorphous cores [58]. High temperatures (>200 °C) promote graphitization and enhance fluorescence in aggregated particles. Reaction time influences the extent of carbonization. Shorter durations may yield partially carbonized products, whereas prolonged processing time can lead to over-carbonization with larger-sized particles [59]. In solvothermal carbonization, pressure is always autogenous and is determined by the vapor pressure of the solvent and the carbonization temperature. Sometimes, pressure can affect reaction kinetics and carbonization mechanisms [55].

Doping with heteroatoms is an effective method for producing CDs with tailored properties, especially bioactivity. Nitrogen doping improves quantum yield and influences antioxidant and antimicrobial activities. Sulphur doping improves RSA, while phosphorus doping alters electronic and surface acid–base properties. Phenolic doping improves RSA and antimicrobial properties. Co-doping with multiple heteroatoms often produces synergistic effects. Surface passivation with agents such as polyethylene glycol not only increases fluorescence efficiency but also enhances toxicity and complexity in regulatory acceptance [60].

### 4.3. Properties of Carbon Dots

#### 4.3.1. Structural and Morphological Characteristics

**Figure 4:**
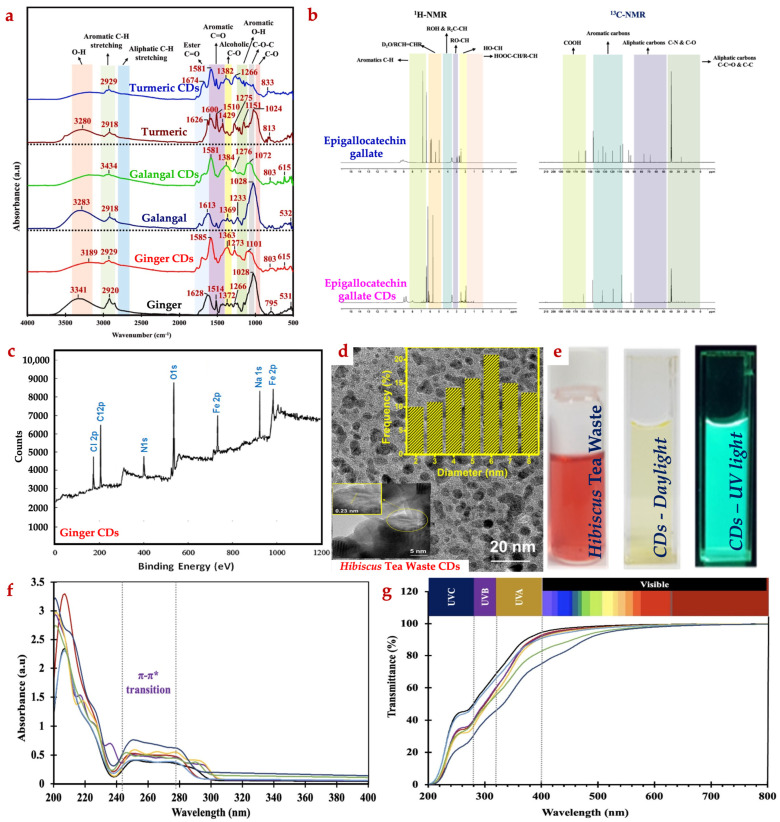
(**a**) FTIR spectra of CDs from ginger, galangal, and turmeric peels. Adapted from Ponnusamy, Buatong, Prodpran, Kim, Rhim and Benjakul [10]. (**b**) ^1^H and ^13^C NMR spectra of CDs from epigallocatechin gallate. Adapted from Ponnusamy, Murugan, Mittal, Saetang, Prodpran, Rhim and Benjakul [15]. (**c**) XRD pattern of ginger-derived CDs. Adapted from Abd-El-Nabey et al. [61]. (**d**) TEM image, including diameter histogram, and (**e**) visual appearance under daylight and UV light of *Hibiscus*-derived CDs. Adapted from Mohandoss et al. [62]. (**f**) UV–Vis absorption spectra and (**g**) UV-blocking performance of tuna stick water-derived CDs. Adapted from Ponnusamy et al. [63].

CDs exhibit numerous structural features, including fluorescence and UV-barrier properties, that are beneficial for active food packaging applications. These properties arise from their nanoscale dimensions and molecular-level surface chemistry (Figure 4).

The surface chemistry of CDs, as confirmed by Fourier transform infrared spectroscopy (FTIR), commonly shows the presence of surface-functionalized groups such as hydroxyl (–OH), COO^−^, carbonyl (CO), and amine groups (Figure 4a). The FTIR spectra revealed that hydrothermal synthesis converted the complex precursor into quantum-sized CDs by breaking down the aliphatic domains. The formed CDs exhibited the enhanced polarity due to the dominance of –OH and C=O groups on their surfaces, while the aromatic nature of the precursors was partially preserved. These structural changes plausibly determine the functional properties of CDs, such as improved solubility, compatibility with hydrophilic or hydrophobic biopolymers, reactivity toward food components, and ability to inactivate microorganisms [15].

Nuclear magnetic resonance (NMR) spectroscopic characterization of CDs indicated that CDs possess structures that are distinct from their precursors (Figure 4b). After hydrothermal carbonization, several peaks in the aromatic carbon region disappeared or became weakened, indicating that the basic structure of the precursors was disrupted. Meanwhile, multiple resonance peaks appeared in the aliphatic region, suggesting the formation of various COO^−^ functional groups. Combined with FTIR spectra, this typical characteristic was attributed to an increase in *π*–*π* stacking interactions of the aromatic rings during the formation of the CDs core. Additionally, the surface of the CDs was covered with polar functional groups attached to aliphatic carbons. New peaks of varying intensities were observed in the range of 20–40 µg/mL, corresponding to aliphatic carbons bonded with –OH and C=O groups on the carbonic core formed during carbonization. Based on ^13^C–NMR analysis, certain changes occurred during the synthesis process, indicating the formation of highly polar functional groups such as –OH and C=O on the CDs surface. The ^13^C–NMR spectra also showed an increase in the peak intensity of aromatic carbons, along with the appearance of new carbon peaks in the aliphatic and C=O regions [15]. X-ray photoelectron spectroscopy demonstrated the presence of carbon in several forms, such as C–C, C–O, C=O, and O–C=O (Figure 4c) [7].

Transmission electron microscopic (TEM) images of CDs revealed spherical or near-spherical particles for most agro-waste-derived CDs (Figure 4d). The internal structure varied depending on the source material, carbonization technique, and synthesis conditions. Some CDs displayed partially crystalline cores with lattice spacings characteristic of graphitic carbon, whereas agro-based CDs appeared predominantly amorphous with minimal crystalline regions. CDs synthesized from agro-waste, such as fruit peels and crop residues, typically have sizes in the range of 8–10 nm, while CDs derived from CS possesses the higher size (10–15 nm). The differences were plausibly due to their varying molecular structures and carbonization conditions [10].

#### 4.3.2. Color, Optical Properties and Photoluminescence

Agro-waste-derived CDs typically appear brown or yellow-brown in aqueous solution (Figure 4e). The color intensity depends on concentration, precursor type, and synthesis conditions [18]. This inherent coloration is generally considered for packaging applications, as it can influence the appearance of packaged seafood.

The UV–Vis absorption spectra of CDs typically exhibit characteristic features, including two distinct peaks (Figure 4f). The primary peak appears in the 200–300 nm range, corresponding to *π*–π* transitions associated with aromatic sp^2^ carbons, such as aromatic C=C bonds and conjugated double bonds (–C=C–). Additionally, a secondary shoulder peak in the 300–400 nm range is attributed to *n*–π* transitions associated with C=O bonds in the carbon cores [12]. These peaks highlight the electron-donating effects of the multiple –OH groups, which enhance absorption intensity. Compared to the precursors, the peak intensity of the CDs is relatively higher, and this may be attributed to the exposure of aromatic compounds after hydrothermal carbonization. Strong absorption in the UV region gradually decreases into the visible range, consistent with transitions observed in the structural makeup of CDs [64]. UV-blocking capability helps protect light-sensitive nutrients and lipids from photodegradation [10] (Figure 4g).

Photoluminescence excitation (PLE) spectra provide the information on the energy levels of CDs associated with emission bands ranging from blue to red. Upon excitation with UV light, CDs emit fluorescence at longer wavelengths. Functional groups such as –OH, –COOH, and C=O drastically influence PLE by altering surface energy states that serve as radiative recombination centers.

Quantum confinement effects and interactions between the sp^2^ carbon core and surface groups further modulate optical properties, enabling tunable PLE. The CDs exhibiting higher PLE intensity show the enhanced light interaction and more efficient emission [7]. Quantum yield values of agro-waste-derived CDs are often moderate (approximately 1–20%) but can be improved via heteroatom doping. The differences in quantum yield might arise from variations in the carbon core structure and the presence of nitrogen- or sulfur-containing functional groups, which are known to enhance fluorescence [65]. The relatively lower quantum yield could be caused by poor carbonization process or numerous surface defects that promote non-radiative recombination.

#### 4.3.3. Antioxidant Activity

**Figure 5:**
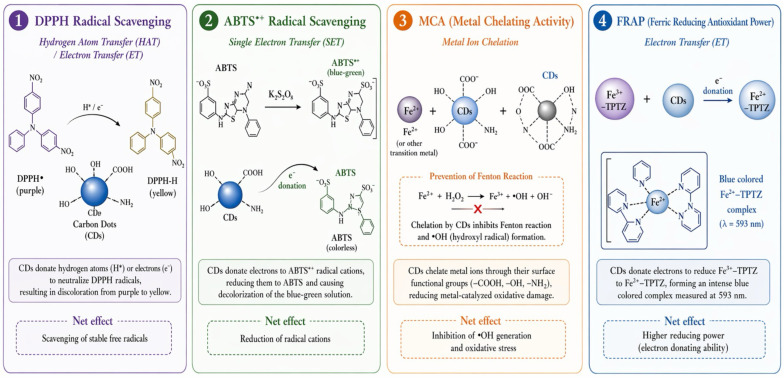
Schematic mechanism of antioxidant activity of agro-waste-derived carbon dots.

CDs can mitigate oxidation through several complementary pathways. The dominant mechanism is free RSA (Figure 5). CDs generally show higher ABTS radical scavenging activity (ABTS-RSA) than DPPH radical scavenging activity (DPPH-RSA) [14]. This disparity is common and can be attributed to the different reaction mechanisms and radical species involved in the two RSA assays. The ABTS^+^ radical cation is more readily scavenged by electron-donating groups in an aqueous system, while the DPPH• radical is a stable, non-ionic radical requiring direct hydrogen atom transfer, especially in less polar systems [14]. The surface functional groups, such as –OH, C=O, and NH_2,_ of the CDs might stabilize the ABTS^+^ by donating electrons [12]. The CDs also showed excellent metal-chelating and ferrous-reducing power due to their multiple –OH groups and aromatic rings, which effectively chelated metal ions [11]. CDs can inhibit metal-catalyzed oxidation processes, such as the Fenton reaction, that take place in perishable foods prone to oxidative deterioration [66]. Surface functional groups such as –OH and COO^−^ moieties, along with conjugated *π*-systems within the carbon core, can donate electrons or hydrogen atoms to neutralize free radicals and terminate oxidative chain reactions [56].

Nitrogen doping often further improves antioxidant properties by increasing electron density and facilitating redox reactions [67]. Antioxidant activity usually increases with CDs concentration, up to an optimal level. Beyond this concentration, some systems may exhibit pro-oxidant behavior, particularly in the presence of transition metals, underscoring the need for optimization of the concentration used in real food matrices [14].

#### 4.3.4. Antimicrobial Activity

**Figure 6:**
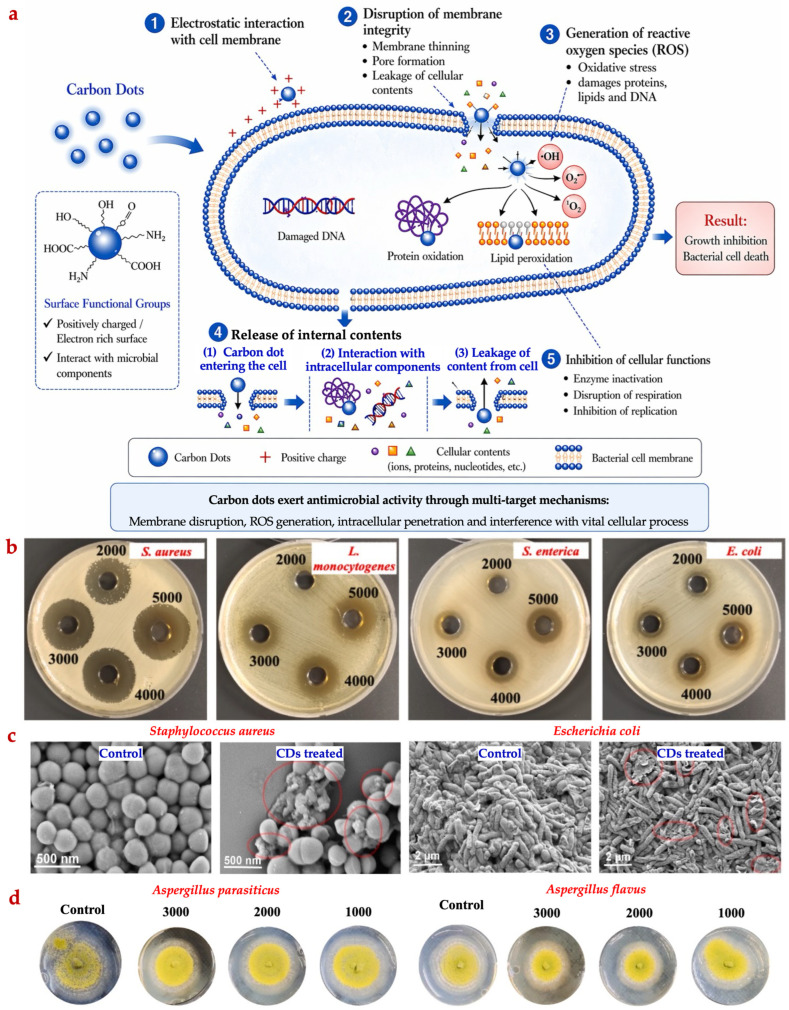
(**a**) Schematic mechanisms of antimicrobial activity of agro-waste-derived carbon dots (CDs). (**b**) Antibacterial activities of mango peel-derived CDs. Adapted from Ponnusamy, Khan, Prodpran, Kim, Benjakul and Rhim [7]. (**c**) SEM images of *Staphylococcus aureus* and *Escherichia coli* treated with curcumin-derived CDs. Adapted from Abbas et al. [68]. (**d**) Antifungal activity of galangal-derived CDs against *Aspergillus parasiticus* and *A. flavus*. Adapted from Ponnusamy, Buatong, Prodpran, Kim, Rhim and Benjakul [10].

CDs also exhibit antimicrobial activity against a wide range of microorganisms, and several mechanisms contribute to their antimicrobial effects (Figure 6) [13].

CDs are known to interact with bacterial cell surfaces, thereby altering the electrochemical potential between positively charged CDs surfaces and negatively charged bacterial cell envelopes and reducing membrane integrity [11]. This interaction can lead to hyper-acidification of the cytoplasm via proton donation, thereby interfering with crucial metabolic pathways. When CDs penetrate the bacterial cell membrane, cell proteins are damaged, and internal cytoplasmic components are released, resulting in cell death (Figure 6c) [69]. Chromosomes condense, followed by disruption of DNA replication take place, and gene expression is also disrupted. Due to their quantum-sized nature, CDs can readily penetrate bacterial cell walls, regardless of bacterial type, thereby enhancing their oxidative capacity and inhibiting enzymes. This leads to bacterial cell rupture, oxidative stress, and eventual cell death [10]. CDs can disrupt bacterial cell walls and produce ROS through photoactivation. When exposed to visible or natural light, CDs interact with bacterial cells, stimulating the generation of –OH free radicals and singlet oxygen in water or air. ROS generation leads to lipid oxidation, membrane disruption, mitochondrial dysfunction, and apoptosis, ultimately resulting in bacterial cell death [11].

#### 4.3.5. Cytotoxicity and Biocompatibility Assessment

Despite the use of natural precursors, CDs for food-contact applications require thorough safety evaluation. Cytotoxicity and biocompatibility must be assessed using relevant in vitro and, ultimately, in vivo models. CDs exhibited lower toxicity toward BJ cells and HDFa cells, with high cell viability. Based on the MTT assay, HDFa cells exposed to CDs at 200 µg/mL detached from the substrate and underwent apoptosis, highlighting the sensitivity of mitochondrial proteins to CDs at high concentrations, as the MTT assay specifically measures mitochondrial dehydrogenase activity [70]. CDs at low concentrations are biocompatible, making them suitable for application in biological systems and sensitive cellular environments. Their small size and surface functionalization also enable efficient cellular uptake without causing significant oxidative stress or structural damage to cellular components [14]. This biocompatibility supports their potential for biomedical applications such as targeted drug delivery, bioimaging, and biosensing. CDs can integrate smoothly with cellular organelles without adversely affecting mitochondrial function or membrane integrity [71].

The cytotoxicity of CDs is largely governed by their surface chemistry and physicochemical properties. Surface functional groups, such as C=O and –OH, can bind to thiol groups in mitochondrial proteins, disrupting mitochondrial function and leading to cell toxicity. At higher concentrations, CDs may aggregate, further increasing cytotoxic effects by compromising cellular membranes [72]. When CDs interact with cells under light or oxygen exposure, ROS production is enhanced, leading to damage to nucleic acids, enzymes, and organelles such as mitochondria. This oxidative stress can trigger apoptosis or necrosis, depending on the severity of the cellular damage [73]. The size of CDs also plays a key role in biological interactions, with smaller particles showing higher cellular uptake and potentially greater cytotoxic effects [70].

Although CDs are generally considered to have low cytotoxicity, their effects can vary depending on experimental conditions and cell type [72]. Higher cytotoxicity has been reported at elevated concentrations or with prolonged exposure, though such conditions typically do not occur in imaging or therapeutic applications. This variability highlights the importance of well-controlled CDs surface properties in reducing toxicity while preserving their functional benefits for food and biomedical applications [7].

Most agro-waste-derived CDs show low cytotoxicity [72]. The genotoxicity tests generally suggest minimal DNA damage for well-synthesized and purified CDs, although the data remain limited and must be expanded before regulatory acceptance [74]. Overall, available scientific evidence indicates that many agro-waste-derived CDs can be engineered to exhibit favorable safety profiles, particularly when anticipated migration levels from packaging into food are comparatively low and further diluted within the food matrix. Nonetheless, a systematic toxicological assessment conducted in accordance with established regulatory guidelines is essential for commercial deployment.

### 4.4. Enhancement of Bioactivities by Doping with Active Compounds

The bioactivities of agro-waste-derived CDs can be further enhanced by doping with functional elements or molecules while adhering to green chemistry principles. Doping strategies are employed to enhance specific properties, such as fluorescence, antioxidant capacity, or antimicrobial potency [75].

Nitrogen doping is the most extensively studied approach. Incorporation of nitrogen atoms into the carbon framework introduces additional energy levels, often increasing fluorescence quantum yield. Nitrogen-containing functional groups, including amine and amide moieties, can also improve electron-donating ability and surface charge, thereby enhancing both antioxidant and antimicrobial activities [66]. Common nitrogen sources include urea, ammonium hydroxide, amino acids, and nitrogen-rich biomasses.

Sulphur doping modifies CDs properties by introducing larger atoms that distort the carbon lattice and generate new surface functionalities (e.g., thiol and sulfonate groups). This can improve radical-scavenging performance and tune surface chemistry. Typical sulphur sources include cysteine and thiourea [76]. Phosphorus doping alters electronic characteristics and surface acidity, potentially enhancing catalytic or adsorption-based functions. Phosphoric acid, phytic acid, and phosphate salts are often used to incorporate phosphorus [77].

Metal doping with selected elements such as iron, zinc, or copper can impart additional functionalities, including enzyme-mimicking (peroxidase-like) activity or enhanced antimicrobial effects. However, metal-containing systems raise particular concerns regarding metal leaching and toxicity in food-contact contexts and therefore require stringent safety evaluation [78].

Doping with natural polyphenols (e.g., gallic acid, catechin, quercetin, tannic acid) represents a particularly attractive green strategy. Polyphenols provide the intrinsic antioxidant activity and, in some cases, antimicrobial properties. They can be integrated into the CDs structure or onto the surface [15]. Similarly, amino acid doping introduces both nitrogen and specific side-chain functionalities; for example, cysteine provides simultaneous nitrogen and sulphur; lysine provides additional amine groups; and arginine offers guanidinium functionality [66,79]. Organic acids such as citric acid and ascorbic acid can enhance fluorescence and antioxidant capacity, respectively, while chelating agents like EDTA may modulate interactions with metal ions [80].

Co-doping with multiple elements or compounds often leads to synergistic effects. Nitrogen–sulphur co-doping combines the respective benefits of each heteroatom, while nitrogen–phosphorus co-doping can further improve fluorescence and alter surface reactivity [66]. Dopant concentration is a critical variable; there are usually optimal levels for achieving desired properties for each dopant employed. Excessive doping can decrease quantum yield, alter particle stability, or introduce toxicity [81]. Consequently, systematic optimization is required for each CD–dopant combination, especially for food-related applications.

## 5. Effect of Carbon Dots on Mechanical and Barrier Properties of Packaging Films

### 5.1. Mechanical Properties

The mechanical properties, including thickness, tensile strength, and elongation at break, of the packaging films determine their ability to protect food during handling, transportation, and storage. The incorporation of CDs significantly affects these characteristics. The packaging film’s thickness showed a negligible difference when CDs were added, likely due to the small size and hydrophilic nature of the CDs, which promoted uniform dispersion within the film matrix [7]. Tensile strength (TS) of the films increased substantially, suggesting that CDs could serve as reinforcing agents to some extent, thereby improving film integrity. The strengthening mechanism involves multiple factors. Nano-sized CDs possess high surface area-to-volume ratios. Their surfaces interact with polymer chains through hydrogen bonding, electrostatic interactions, and van der Waals forces. These interactions create physical cross-linking within the polymer matrix [14]. Generally, the optimal concentration ranges from 1% to 5% (*w*/*w*). Beyond these levels, excessive CDs may aggregate, and the intensive polymeric chain interactions likely reduce tensile strength [16]. Young’s modulus measures film stiffness or rigidity, and it indicates the resistance of the films to elastic deformation. Higher modulus values correspond to stiffer films, and the incorporation of CDs generally increases Young’s modulus through the same reinforcing mechanisms. The increase in modulus reflects the formation of a more rigid polymer–CDs network. The stiffening effect is particularly pronounced at temperatures above the polymer glass transition, where CDs restrict chain mobility [7].

Elongation at break (EAB) measures film flexibility and extensibility. High elongation is desirable for seafood packaging to withstand deformation during handling. The effect of CDs on EAB varies with CDs type and concentration. Moderate CDs loadings in biopolymer-based films often maintain or reduce elongation. Cross-linking increases interactions between polymeric chains, thereby restricting their mobility [16]. However, CDs with appropriate surface functionality can sometimes enhance elongation through plasticizing effects. The balance between strengthening and flexibility requires optimization between CDs incorporation and the concentration of plasticizer commonly used for each application. The mechanical property improvements depend on the solvent in which CDs are dispersed and the compatibility between the polymers and other film components. Uniformly distributed nanoparticles provide a homogeneous film with consistent reinforcement throughout the film network. Aggregated particles create stress concentration points, which affect thickness and mechanical properties [82].

Cellulose nanofiber/pullulan films incorporated with avocado peel-derived Zn-doped CDs showed the most pronounced mechanical enhancement, in which TS increased by 45% and EAB increased by 64% at 5% *w*/*w* loading [79]. This is attributed to the high surface reactivity of Zn-doped CDs, which likely form strong hydrogen bonds and induce crosslinking with the hydroxyl-rich cellulose and pullulan chains. Similarly, tangerine peel-derived N-doped CDs improved the TS of chitosan–pullulan films by 22% [83]. The magnitude difference between the avocado and tangerine systems may stem from the different CDs surface chemistry and the initial polymer blend. In contrast, cumin powder-derived CDs combined with Ti–MOFs increased the TS of carboxymethyl cellulose/agar films by 17% [78]. The relatively low enhancement, despite titanium doping, suggests that the CD–MOF hybrid may form rigid, poorly dispersible aggregates, thereby limiting effective stress transfer. Crayfish shell-derived N-CDs in konjac glucomannan/sodium alginate films had enhanced mechanical and barrier properties [84].

Furthermore, the hydrothermal synthesis technique produces CDs with oxygen- and nitrogen-rich functional groups that can bond to polymer matrices via hydrogen bonding. However, the mechanical properties of CD-biopolymer composites are highly dependent on precursor sources, doping elements, and polymer matrix compatibility. Several peels contain flavonoids and pectin, which can yield CDs with CO and –OH groups, whereas animal-derived waste yields CDs with amine functionalities. These differences directly determine the efficiency of mechanical reinforcement.

### 5.2. Barrier Properties

Barrier properties of the film determine its effectiveness in protecting food from oxygen, water vapor, and light. The incorporation of CDs significantly enhances these barrier characteristics. Water vapor permeability measures the rate at which water vapor passes through the film. This property is critical for seafood packaging, as moisture loss causes quality deterioration, while excessive moisture can accelerate spoilage. Biopolymer films are hydrophilic compared to conventional plastics, which exhibit high water vapor barrier properties [85]. CDs can either decrease or increase the water vapor barrier property, depending on the type and level of CDs used in the film. The incorporation of some CDs substantially increased water vapor permeability. The inherent hydrophilicity of CDs led to hydrogen bond formation with water molecules. This could disrupt the film’s compactness and rigidity, leading to high moisture permeability. However, some CDs with highly hydrophobic surface domains may also repel water, creating regions of reduced permeability. Therefore, it was confirmed that permeability reduction depends on CDs concentration, hydrophilicity, polymer constituents, and dispersion [86].

Oxygen and carbon dioxide permeability measure the transmission rate of these gases through the film. Gas transmission through the packaging material is critical for preventing lipid oxidation and microbial growth in seafood. Biopolymer films based on GL generally exhibit a good oxygen barrier [87]. The incorporation of CDs further improves gas barrier properties through cross-linking with the polymers, creating a tortuous path for oxygen molecules [88]. CDs can also scavenge oxygen entering the film, providing active barrier functionality beyond passive resistance. Improving the oxygen barrier is particularly valuable for fatty fish, which are highly susceptible to oxidation. Reduced oxygen transmission can, to some extent, lower lipid oxidation and extend the shelf life of fatty fish, including salmon, mackerel, and tuna [89].

Crayfish shell N-CDs preserved crayfish meat for 8 days at 4 °C [84], and purple kohlrabi peel ZnO-CDs extended shrimp shelf life with visible spoilage indication [86]. Therefore, these CDs could improve stability, mainly owing to their antimicrobial activity. Nonetheless, efficacy was governed by several factors, especially the source of CDs and the active components used in combination for synergism.

### 5.3. Optical Properties

Optical properties influence both film appearance and food protection. Transparency affects consumer acceptance, since many consumers prefer to see the packaged products. Light barrier properties protect light-sensitive food components from degradation or decomposition. Transparency and opacity determine how clearly the packaged food can be visualized. High transparency is desirable for many seafood products, allowing visual inspection without opening packages [85]. CDs incorporation may slightly reduce transparency, particularly at higher concentrations. The reduction in transparency occurs through light scattering by particles. The small size of CDs minimizes scattering, thus preserving reasonable transparency even at functional concentrations [88]. Most CDs incorporated films maintain adequate transparency for commercial acceptance.

Color changes may occur with the incorporation of CDs. Agro-waste-derived CDs typically appear brown or yellow-brown and dark with an upsurge in CDs concentrations [14]. When incorporated into films, they impart slight coloration. The color intensity depends on the CDs source, concentration and intrinsic color. The UV light barrier showed a significant improvement upon incorporating CDs, achieving nearly 99% blocking of UV-C and UV-B. These properties are critical for protecting lipid-rich seafood, such as clams [18]. The lipids in clams are abundant in PUFAs, which are highly vulnerable to degradation via photo-oxidation, a process primarily initiated by UVB radiation (280–320 nm). CDs strongly absorb UV radiation through their conjugated carbon cores. The UV blocking ability derives from electronic transitions within CDs structures, and even low CDs loadings provide substantial UV protection [18]. Based on light transmission spectra across the UV and visible ranges, CDs exhibited barrier properties in the UV but showed increased transmission in the visible. CD-enhanced films typically show near-complete UV blocking while maintaining useful visible transmission.

UV barrier performance is consistently enhanced across all CD-incorporated films, but the degree of protection varies notably. For instance, nitrogen-phosphorus co-doped CDs from green tea achieved 93% UV-A and 99% UV-B blocking in CS/starch films [90]. In the case of titanium dioxide-doped CDs from sweet potato peel, complete (100%) UV protection was achieved in carrageenan-based films [75]. Interestingly, even without doping, nitrogen-doped CDs from crayfish shell [84] and tangerine peel [83] still blocked 98–100% of UV-B radiation, suggesting that nitrogen contributes to their high effectiveness in the UV-B range.

### 5.4. Thermal Stability and Degradation Behavior

CDs incorporation affected the thermal behavior of the films. CDs showed improved thermal stability when incorporated into biopolymer films. CDs loading typically increased the thermal degradation temperature in the initial phase (60–80 °C). This could plausibly be due to interactions between CDs and the polymer matrix, as evidenced by reduced moisture absorption [18]. The nanoparticles create physical barriers that delay the escape of volatile decomposition products. Chemical interactions between polymer chains might be disrupted by the incorporation of CDs, leading to char formation that could act as a thermal barrier. CDs-incorporated films generally showed delayed degradation and increased char residues compared to control films without CDs incorporation. CDs incorporation increased the glass transition temperature by restricting polymer chain mobility, and this thermal shift depends on the CDs–polymer interactions [9].

The melting behavior of semi-crystalline biopolymers was affected by CDs, as evidenced by the changes in melting temperatures and enthalpies. CDs plausibly acted as nucleating agents and promoted crystallization during film formation, thereby reducing crystallinity. These changes in crystallinity improved barrier and mechanical properties by creating more ordered regions with reduced permeability. However, excessive crystallinity may reduce EAB [57]. Most agro-waste-derived CDs showed good thermal stability. However, the advanced characterization, such as dynamic mechanical analysis and XRD crystallinity changes, should be performed more extensively. These observations are required to elucidate the mechanical behavior and thermal transitions of these polymers [18].

## 6. The Use of Carbon Dots-Incorporated Films for Seafood Preservation

### 6.1. Antimicrobial Efficacy Against Seafood Spoilage Microorganisms

The antimicrobial activity of CDs-enhanced films represents a crucial property for seafood preservation. Seafood spoilage microorganisms, particularly *Pseudomonas* spp., *Shewanella* spp., and *P. phosphoreum*, are effectively inhibited by CDs incorporated into biopolymer films [57]. The antimicrobial efficacy varies with CDs characteristics, including particle size, surface charge, and functional group composition, depending on the source and dopants. The persistence of antimicrobial activity in film matrices ensures the sustained protection throughout storage, with surface-localized CDs inhibiting microbial growth and biofilm formation. The spectrum of antimicrobial activity of CDs extends beyond spoilage organisms to some pathogens. *Listeria monocytogenes*, *Vibrio parahaemolyticus*, and *Salmonella* spp. are inhibited by CDs-containing films in various studies.

Table 2 demonstrates the broad-spectrum antimicrobial efficacy of various CDs formulations. Nitrogen-doped CDs derived from crayfish shells incorporated into konjac glucomannan and sodium alginate films exhibited excellent photodynamic antibacterial effects against *S. putrefaciens* (99.2%) and *Staphylococcus aureus* (98.9%) [84]. *S. putrefaciens* is a dominant spoilage organism in marine seafood, responsible for TMA production and characteristic fishy odors. Nitrogen-phosphorus co-doped CDs from green tea, incorporated into CS and ST films, exhibited strong antibacterial activity against *L. monocytogenes*, *Escherichia coli*, and *S. aureus* [90]. The films maintained bacterial counts below 2.5 log CFU/g in wrapped meat after 48 h at 20 °C, demonstrating the effectiveness of the films. Zinc oxide-doped CDs from purple kohlrabi peels incorporated into carrageenan-based films with anthocyanin achieved complete inhibition of *L. monocytogenes* and reduced *E. coli* by 8.1 log CFU/mL after 12 h [86]. The films also maintained freshness, which could be monitored by visible color changes in shrimp. In general, color changes were observed in response to the pH of shrimp. When L-cysteine-modified CDs from Duea ching were incorporated into CS and GL films, antibacterial and antifungal activities, along with excellent UV barrier properties, were observed. The films maintained total viable counts below the spoilage limit of 6 log CFU/g in Asian seabass slices after 15 days of refrigerated storage [17].

### 6.2. Antioxidant Activity and Lipid Oxidation Inhibition

Lipid oxidation represents a major deterioration reaction in seafood, particularly for fatty species. CD-containing films address this challenge through multiple antioxidant pathways, primarily RSA facilitated by surface functional groups, including –OH, COO^−^, and CO groups, as well as electron-rich conjugated carbon cores [18].

Peroxide value measures primary oxidation products, hydroperoxides, in seafood during storage. Thiobarbituric acid reactive substances (TBARS) measure secondary oxidation products, particularly malondialdehyde, which are responsible for rancid and fishy off-odor. Hexanal and other volatile aldehydes serve as specific markers for the oxidation of particular fatty acids. The antioxidant ability of CDs is associated with the prolonged shelf life of seafood. The suppression of lipid oxidation has been demonstrated by reduced hydroperoxide formation in the early stages and lower TBARS accumulation in the middle or final stages. Synergistic effects between CDs and the inherent antioxidants in seafood may occur. Seafood contains endogenous antioxidants, including tocopherols and carotenoids [2].

As summarized in Table 2, various CDs formulations demonstrate significant antioxidant activity across different assay systems. Nitrogen-doped CDs derived from crayfish shells achieved 77.9% DPPH scavenging when incorporated into konjac glucomannan and sodium alginate films, while UV transmittance at 300 nm was reduced to 16.9% [84]. This UV-blocking capability is particularly important for protecting light-sensitive PUFA from photooxidation. Nitrogen-phosphorus co-doped CDs from green tea, incorporated into CS and ST films, exhibited strong antioxidant activity, with 98.0% ABTS-RSA and 71.4% DPPH-RSA [90]. The films also achieved exceptional UV blocking of 93.1% for UV-A and 99.7% for UV-B, providing comprehensive protection against light-induced oxidation. *Sophora flavescens* root-derived CDs co-doped with titanium dioxide and nitrogen demonstrated the highest antioxidant activity, achieving 100% ABTS and 71.1% DPPH scavenging when incorporated into CS films [91]. UV blocking reached 99.9% for UV-B and 99.6% for UV-A, extending the shelf life of sliced bread to 12 days. Cumin powder-derived CDs combined with Ti-MOF achieved 100% ABTS and 57.8% DPPH scavenging, with UV blocking of 95.7% (UV-B) and 84.7% (UV-A) [78].

Zinc-doped CDs from avocado peel exhibited 100% ABTS-RSA and 68% DPPH-RSA with complete UV blocking at 5% (*w*/*w*) loading [79]. L-cysteine-modified CDs from Duea ching, when incorporated into CS and GL films, blocked 97.1% of UV-B radiation and significantly reduced chemical deterioration in Asian seabass slices [17]. The antioxidant mechanisms involve both direct RSA and indirect protection through UV absorption. CDs from polyphenol-rich precursors, including green tea and purple kohlrabi peels, showed enhanced RSA, probably due to the functional groups of the incorporated phytochemicals. Nitrogen doping generally enhances antioxidant activity through electron donation, whereas co-doping with phosphorus or sulfur yields synergistic effects. The combination of antioxidant activity and UV blocking in CD-enhanced films provides comprehensive protection against lipid oxidation, effectively retarding rancidity and preserving the nutritional quality of seafood during storage. CDs strongly absorb UV radiation. This blocking prevents photooxidation initiation, which is particularly important for seafood displayed under retail lighting. The combination of UV barrier and RSA provides dual protection, augmenting preservation potential.

### 6.3. Applications of CDs-Based Packaging in Various Seafood

The effectiveness of CDs-incorporated films across diverse seafood products has been demonstrated, with preservation outcomes varying by seafood type, CDs formulation, film matrix, and storage conditions. Nitrogen-doped carbon quantum dots derived from crayfish shells, when incorporated into konjac glucomannan and sodium alginate films, preserved crayfish meat for up to 8 days at 4 °C [84]. L-cysteine-modified CDs from Duea ching, incorporated into CS and GL films, blocked 97.1% of UV-B radiation. Asian seabass slices wrapped with films containing 5% modified CDs maintained total viable counts below the spoilage limit of 6 log CFU/g [17]. Zinc oxide-doped CDs from purple kohlrabi peels incorporated into carrageenan-based films with anthocyanin demonstrated effective freshness monitoring through visible color changes, reduced spoilage rates, and extended shelf life, with distinguishable colorimetric responses across pH 7–12 buffers and to volatile ammonia, enabling real-time spoilage indication [86]. Similarly, titanium-doped CDs from sweet potato peel in carrageenan-based pH-sensing films with anthocyanin retarded quality deterioration while serving as effective indicators of shrimp spoilage [75]. Shelf life extensions ranged from several days to more than a week, depending on the type of seafood and storage conditions. Crayfish was preserved for 8 days at 4 °C [84], and chicken and tofu had reduced microbial growth over 9 days at 10 °C [79].

**Figure 7:**
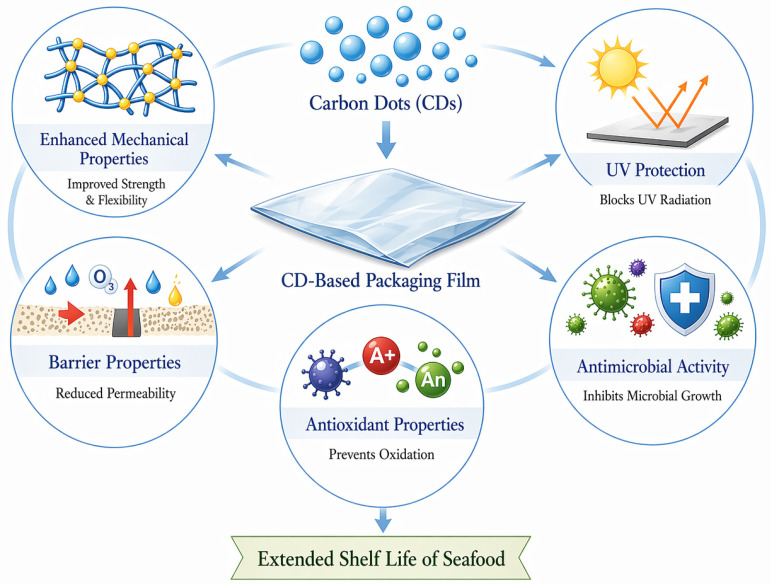
Schematic illustration of carbon dots incorporated into packaging films and their properties and bioactivities, leading to shelf life extension of seafood.

The effectiveness of CDs-based packaging depends on CDs concentration, biopolymer matrix selection, and seafood type; fatty fish require greater antioxidant protection, while lean fish and shellfish primarily benefit from CDs-containing films through antimicrobial activity. The integration of intelligent features, including pH sensing and ammonia detection, enables real-time freshness monitoring, positioning CDs-based packaging as a promising technology for reducing seafood waste and ensuring product quality throughout the supply chain (Figure 7).

## 7. Safety Assessment, Migration Behavior, and Regulatory Considerations

The safety of CDs for food-contact applications requires a comprehensive evaluation, even when natural agro-wastes are used as the starting material. The nanoscale dimensions and surface chemistry of CDs pose potential toxicological concerns that must be addressed through systematic testing in accordance with established guidelines. Table 3 summarizes cytotoxicity studies of agro-waste-derived CDs across various cell lines, providing important insights into their biocompatibility. In vitro cytotoxicity studies provide the initial safety data using mammalian cell lines. CDs derived from *Elaeis guineensis* leaves via pyrolysis exhibited selective cytotoxicity toward HeLa cervical cancer cells, with an IC_50_ of 468.1 µg/mL, while remaining biocompatible with HS27 human fibroblasts, suggesting selectivity between cancerous and normal cells [94]. Wet olive pomace-derived CDs synthesized hydrothermally showed high viabilities in both L929 mouse fibroblasts and HeLa cervical cancer cells up to 500 µg/mL, with mild toxicity (78% viability for L929 and 64% for HeLa) observed only at the higher concentration of 1000 µg/mL [95].

CDs derived from turmeric, ginger, and galangal peels at concentrations up to 200 µg/mL showed cell viability exceeding 80% in normal human fibroblast BJ cells, indicating good biocompatibility [10]. Enoki mushroom-derived CDs exhibited negligible cytotoxicity in mouse fibroblast L929 cells, with cell viability exceeding 95% across concentrations ranging from 0.1 to 0.5 mg/mL [42]. L-cysteine-modified CDs from Duea ching showed above 80% viability at concentrations of 31.3 and 62.5 µg/mL in normal human fibroblast BJ cells, suggesting low cytotoxicity [17].

Dried German chamomile flowers and residual biomass-derived CDs exhibited high biocompatibility, with >88% viability at 1000 µg/mL in normal human fibroblast BJ cells [39]. Oil palm biomass-derived CDs were reported as nontoxic to Vero cells even at high concentrations [94]. These findings collectively demonstrate that most agro-waste-derived CDs show minimal cytotoxicity at concentrations relevant to packaging applications, with IC_50_ values typically exceeding the expected migration levels.

In vitro cytotoxicity studies using mammalian cell lines, including human intestinal Caco-2 cells and hepatic HepG2 cells, confirmed preliminary safety, as assessed by MTT, MTS, and CCK-8 assays. Numerous agro-waste-derived CDs exhibited negligible toxicity at concentrations below 100 μg/mL [96]. Cytotoxicity is influenced by CDs surface chemistry, with positively charged CDs potentially interacting more strongly with cell membranes [96]. Comprehensive testing following OECD guidelines is needed for commercial formulations. Genotoxicity assessment through Comet assays showed toxicity; upon purification, CDs had minimal genotoxic effects. Acute oral toxicity studies in animal models indicated no significant toxicity at doses up to 1000 mg/kg body weight, while sub-chronic studies spanning 28 to 90 days suggested minimal toxicity [97].

Migration behavior is a critical factor for both safety assessment and functional performance [98]. CDs, which are nanosized particles, have the potential to migrate, although interactions with polymer matrices can occur. Food simulants specified by EU and FDA regulations, including ethanol solutions for aqueous foods and olive oil for fatty foods, are used for migration testing under controlled conditions [99]. Generally, migration levels fluctuate depending on CDs–polymer interactions, film formulation, and the food simulant used. Controlled-release approaches can be deliberately engineered to deliver active compounds to food surfaces with preferred release kinetics. This could be controlled through film formulation, cross-linking, and CDs surface modification [100]. Future approaches require fluorescence spectroscopy, total organic carbon analysis, and mass spectrometry for accurate quantification [15].

Regulatory frameworks for active packaging materials are evolving to address novel CDs. Acceptable doses should be approved by the European Union, in which active substances are subjected to safety evaluation by EFSA under Regulation (EC) No 450/2009 [99]. The novel food regulation may be applicable to CDs if they have not been historically consumed. United States regulations under the Food, Drug, and Cosmetic Act use food-contact notifications as the primary approval pathway, with substances that migrate below 0.5 µg/L potentially eligible for exemption [101]. The EU requires explicit consideration of nanoscale properties in safety assessments. Other jurisdictions, including China, Japan, Canada, and Mercosur countries, have established food-contact material regulations with limited international harmonization, requiring separate evaluations for different markets. Early engagement with regulatory authorities, understanding data requirements for specific jurisdictions, and anticipating questions about nanoscale properties and novel materials are essential for successful commercialization.

**Table 3:** Summary of cytotoxicity of agro-waste-derived carbon dots on various cell lines.

Source of CDs	Synthesis Process	Test System	Concentration	Toxicity Results	Ref
*Elaeis guineensis* leaves	Pyrolysis	HeLa cervical cancer cell line and HS27 human FB cell line	0–2.5 mg/mL	Selective cytotoxicity toward HeLa cervical cancer cells (IC_50_ = 468.1 µg/mL), and biocompatible with HS27 fibroblasts	[94]
Wet Olive Pomace	Hydrothermal	Non-tumoral mouse FB cell line (L929) and a tumoral human cervical cancer cell line (HeLa)	(0.5–1000 µg/mL)	Both cell lines generally showed high viability after 24 h, up to a concentration of CDs of 500 μg/mL. A decrease in cell viability (78% for L929 and 64% for HeLa cells) was only observed at a much higher concentration (1000 μg/mL)	[95]
Turmeric, ginger and galangal peels	Hydrothermal	Normal human FB BJ cells	25–200 μg/mL	Cell viability exceeded 80% up to 200 μg/mL	[10]
Enoki mushroom	Hydrothermal	Mouse FB L929 cells	0.1–0.5 mg/mL	CDs showed negligible cytotoxicity; cell viability >95% at all concentrations	[42]
Duea ching + L-cysteine	Hydrothermal	Normal human FB BJ cells	31.25–250 μg/mL	CDs samples at concentrations of 31.3 and 62.5 μg/mL resulted in greater than 80% viability, suggesting low cytotoxicity	[17]
Chamomile flowers	Hydrothermal	Normal human FB BJ cells	125–1000 μg/mL	Both CDs exhibited high biocompatibility (>88% viability at 1000 µg/mL)	[39]
Oil palm biomass	Hydrothermal	Vero cells	0–30%	CDs derived from oil palm biomass are nontoxic to cells even at high concentrations	[102]

CDs: carbon dots, HeLa: Henrietta Lacks, and FB: fibroblast.

## 8. Current Challenges and Future Perspectives

The translation of CD-enhanced biopolymer films from laboratory scale to commercial application encounters numerous challenges. Current laboratory-scale synthesis methods produce small quantities suitable for research, but industrial production requires large quantities with consistent quality. The design of the hydrothermal carbonization reactor must address heat and mass transfer, as large reactors develop temperature gradients that affect product consistency [103]. Batch-to-batch variability further complicates scale-up practices, since the composition of agro-waste varies with season, source, and storage conditions [104]. This natural variability affects CDs properties and necessitates standardization through precursor blending and processing parameters. Purification methods, including centrifugation and dialysis on an industrial scale, become labour-intensive. Optimization of yield is problematic, as the current CDs yield ranges from 5% to 30%, depending on precursor and method. The diversity of CDs types complicates the comparison between studies [104]. Understanding the molecular mechanisms underlying CDs antimicrobial and antioxidant activities would enable the effective design with a deep knowledge of CDs–polymer interactions and release mechanisms.

Economic viability and market acceptance are equally important [55]. Consumer acceptance requires further studies to reflect the sensory impact on packaged products. The intellectual property landscape of CDs in food packaging still requires careful navigation through freedom-to-operate analyses. However, edible film applications would eliminate packaging waste entirely, although they would require enhanced safety assurance, particularly for those loaded with CDs. Integration with intelligent packaging systems, including QR codes and time-temperature indicators, could further enhance the value of CDs incorporated into active packaging [105]. Addressing these challenges will determine whether CD-containing packaging can be of transition from a promising laboratory concept to a practical commercial reality.

## 9. Conclusions

This review systematically covers the emerging application of agro-waste-derived CDs as functional additives in biopolymer films for active packaging of perishable seafood. Agro-wastes, including fruit peels, leaves, rhizomes, stems, and processing residues from various crops, serve as abundant, low-cost precursors for CDs synthesis. Green synthesis methods, particularly the hydrothermal carbonization approach, convert these wastes into nanoparticles with different functions. This valorization aligns well with CEPs, transforming waste materials into value-added products. CDs possess multiple functional properties and show varying bioactivities depending on their sources. When incorporated into biopolymer matrices, CDs improve multiple film characteristics and enhance bioactivities. The effectiveness of CDs-enhanced films across diverse seafood products has been demonstrated, including suppression of microbial growth and oxidative changes, while maintaining sensory quality for longer periods than conventional packaging. Nonetheless, efficacy depends on the type of seafood and storage conditions. Despite these promising results, several challenges currently limit commercial implementation, including the need for standardized characterization protocols, understanding of migration behavior, and comprehensive toxicological evaluations covering chronic toxicity and bioaccumulation studies. Demonstrating economic viability and market acceptance is also required. Future research directions should focus on developing multifunctional CDs systems, responsive packaging with sensing capabilities, edible film applications, and integration with intelligent packaging technologies. Overall, agro-waste-derived CDs represent a promising nanomaterial platform for developing next-generation active packaging systems that support sustainability goals while addressing seafood preservation needs.

## Figures and Tables

**Table 1:** Carbon dots derived from agro-waste: Precursors, synthesis methods, conditions, properties, and applications.

Agro-Waste Precursor	Synthesis Method	Solvent/Temperature (°C)/time (h)	Size (nm)	Application	Ref
Mango peels	Hydrothermal	Water/200/6	8–10	Active packaging film for shelf life extension of minced pork	[7]
Chitosan	Hydrothermal	Water/200/6	5–15	Active/smart packaging of Pacific white shrimp	[16]
Turmeric	Hydrothermal	Water/200/8	8–10	Bilayer active packaging films for shelf life extension of clams	[18]
Residue of coffee husk extract	Hydrothermal	Water/200/6	1.8–4.2	Shelf life extension of fresh-cut apples (7 days, 4 °C)	[38]
Chamomile flowers	Hydrothermal	Water/200/-	7–10	Active packaging for shelf life extension of a precooked baby clam edible portion.	[39]
Onion peels	Hydrothermal	Water/200/7	1.7–9.0	Antimicrobial effectiveness against key foodborne pathogens and biofilms on common food contact surfaces	[40]
Potato skin	Hydrothermal	Water/200/4	7–10	Gelatin-based active packaging	[41]
Enoki mushroom	Hydrothermal	Water/200/6	8–10	Gelatin/carrageenan-based functional films for active food packaging applications	[42]
Banana peel	Hydrothermal	Water/200/6	8	Chitosan/gelatin-based active packaging	[43]
Turmeric	Hydrothermal	Water/200/5	8	Chitosan nanocomposite films based on photodynamic inactivation technology for antibacterial food packaging	[44]
Spent tea-GQDs	Microwave (100–900 W)	Deionized water/-/3	1.6	Selective probe for Fe^3+^ sensing	
Orange peel-CQDs	Microwave (700 W)	Deionized water/220/0.5	1.16	Photosensitizer for mesoporous TiO_2_ photoelectrodes	[45]
Sago waste	Pyrolysis	-/250–450/1	6–17	Metal ion sensing	[46]
Plant leaves	Pyrolysis	-/250–400/2	-	For sensing, patterning and coding	[47]
Peanut shell	Pyrolysis	-/400/4	8	Visual detection of fluorescence sensitivity for Cu^2+^	[48]

**Table 2:** Summary of agro-waste-derived carbon dots: Sources, doping agents, synthesis methods, and bioactive properties.

Source	Doping Agent	Synthesis Method	Key Findings	Ref
Crayfish shell	Nitrogen	Hydrothermal	Nitrogen-doped CDs incorporated into konjac glucomannan and SA films enhanced mechanical and barrier properties. AO reached 77% DPPH-RSA, while UV transmittance at 300 nm reduced to 16%. The films exhibited excellent photodynamic and AM against *S. putrefaciens* (99.2%) and *S. aureus* (98.9%). No cytotoxicity, and preserved crayfish meat for up to 8 days at 4 °C.	[84]
Green tea	Nitrogen, phosphorus-doped	Hydrothermal	Nitrogen-phosphorus co-doped CDs incorporated into CS/ST films enhanced UV blocking (93% UV-A, 99% UV-B) and AO (98% ABTS, 71% DPPH RSA). The films showed strong AM against *L. monocytogenes*, *E. coli*, and *S. aureus*. Wrapped meat stored at 20 °C maintained bacterial counts below 2.5 log CFU/g after 48 h without significant color change.	[90]
*Sophora flavescens* root	Titanium dioxide- and nitrogen-doped	Hydrothermal	Films showed strong AM against bacteria (*S. aureus*, *L. monocytogenes*, *S. enterica*, *E. coli*) and fungi (*P. chrysogenum*, *A. flavus*). When added to CS films, UV blocking reached 99% (UV-B) and 99% (UV-A). AO was indicated by 100% RSA for ABTS and 71% for DPPH. The films completely inhibited *L. monocytogenes* and *E. coli*, extending sliced bread shelf life to 12 days at 25 °C.	[91]
Cumin powder	Titanium–MOF	Hydrothermal	CDs combined with titanium-based MOFs exhibited strong AO against DPPH and ABTS RSA along with broad-spectrum AM against bacteria and fungi. When incorporated into CMC and agar films, the composite enhanced TS by 17% and improved barrier properties. The films showed strong UV blocking, achieving 95% for UV-B and 84% for UV-A, along with high AO (100% for ABTS and 57.8% for DPPH RSA). AM was demonstrated through complete inhibition of *L. monocytogenes* and significant reduction of *E. coli*. In cherry tomato packaging studies at 4 °C, the films extended shelf life by reducing spoilage.	[78]
Purple Kohlrabi peels	Zinc oxide-doped	Hydrothermal	CA-based active and intelligent packaging films incorporating anthocyanin and zinc oxide-doped CDs exhibited excellent UV blocking ability, blocking 85% of UV-A and 99% of UV-B. AO reached ~99% for ABTS and 58.6% for DPPH RSA. Strong AM was observed, with complete inhibition of *L. monocytogenes* and reduction of *E. coli* by 8.1 log CFU/mL after 12 h of incubation. In shrimp packaging experiments, the films demonstrated effective freshness monitoring through visible color changes, reduced spoilage rates, and extended shelf life.	[86]
Lemon peel	Nitrogen (ethylenediamine)	Hydrothermal	CS films incorporated with nitrogen-doped CDs showed the improved TS, EAB, and UV barrier performance compared to pure CS films. The CS/7% N-CDs composite exhibited high AM, achieving the inhibition of 91% against *E. coli* and 99% against *S. aureus*. In pork preservation studies, samples coated with CS/3% N-CDs films showed significantly lower pH, TVB-N, and TVC. Mold contamination and anthocyanin loss were also reduced, indicating extended shelf life of the food product.	[92]
Sweet potato peel	TiO_2_-doped	Hydrothermal	CA-based active pH-sensing films incorporating anthocyanin and 3% *w*/*w* titanium-doped CDs achieved 100% UV protection, enhanced AO (100% for both DPPH and ABTS RSA), and potent AM with complete eradication of *L. monocytogenes* and *E. coli* after 3 h of incubation. The composite films showed distinguishable colorimetric responses across pH 7–12 buffers and to volatile ammonia. For shrimp packaging, the films retarded the rate of quality deterioration during storage while serving as effective indicators of shrimp spoilage through visible color changes.	[75]
Tangerine peel	Nitrogen	Hydrothermal	Nitrogen-doped CDs incorporated into CS-PL films improved TS by 22%. AO reached 62% for DPPH and 91.6% for ABTS RSA. The films blocked 98% of UV-A and 100% of UV-B while maintaining transparency. AM reduced *L. monocytogenes* by more than 4 log CFU/mL and *E. coli* by more than 5 log CFU/mL. Sliced bread packaged in these films and stored for 12 days at 25 °C and 50% relative humidity maintained excellent quality in terms of appearance, moisture content, hardness, weight loss, and total viable bacterial count.	[83]
Avocado peel	Zinc-doped	Hydrothermal	CDs incorporated into cellulose nanofiber and PL films improved TS by 45% and EAB by 64%. At 5% *w*/*w* loading, the films achieved complete UV blocking (100%), strong AO (100% for ABTS, and 68% for DPPH RSA), and full eradication of *L. monocytogenes* and *E. coli* within 3 h. When applied to chicken and tofu packaging, the films significantly reduced aerobic microbial growth over 9 days at 10 °C without altering product color.	[79]
Duea ching	Nitrogen and sulfur (L-cysteine)	Hydrothermal	CS and GL films containing modified CDs showed enhanced AO and AM properties along with excellent UV barrier, blocking 97% of UV-B radiation. Asian seabass slices wrapped with films containing 5% modified CDs and stored in PP pouches maintained TVC below the spoilage limit of 6 log CFU/g with reduced chemical deterioration over 15 days of refrigerated storage, outperforming samples packed in PP pouches without film wrapping.	[17]
Resveratrol	Nitrogen (o-phenylenediamine)	Hydrothermal	CA and GL films containing nitrogen-doped CDs showed enhanced AO (91%) and AM (98%), along with good biocompatibility (cell viability > 85%). The CDs-incorporated films reduced TVB-N to 11.57 mg/100 g, while higher value was found in the control (23.26). The film could extend shelf life by 5 h at 25 °C and by 24 h at 4 °C, compared to samples without film wrapping. The films also exhibited dual-mode visible and fluorescence color shifts in response to TVB-N-driven alkalization, enabling freshness monitoring.	[5]
Bamboo shoot–shell	Hydrothermal		PL/cellulose containing 5% *w*/*w* CDs showed enhanced AO and AM along with robust UV blocking below 400 nm. Strawberries wrapped with active films and stored under ambient conditions maintained lower TVC, reduced weight loss, and better preservation of acidity, vitamin C, and soluble solids over the storage period, outperforming unwrapped samples.	[93]

CDs: carbon dots, AO: antioxidant activity, AM: antimicrobial activity, SA: sodium alginate, CMC: carboxy methyl cellulose, CA: carrageenan, CS: chitosan, GL: gelatin, PL: pullulan, PP: polypropylene, DPPH: 2,2-Diphenyl-1-picrylhydrazyl, ABTS: 2,2′-Azino-bis(3-ethylbenzothiazoline-6-sulfonic acid), RSA: radical scavenging activity, UV: ultraviolet, MOFs: metal–organic frameworks, TS: tensile strength, EAB: elongation at break, TVB-N: total volatile basic nitrogen, TVC: total viable count, and CFU: colony forming unit.

## Data Availability

No new data were created or analyzed in this study. Data sharing is not applicable to this article.

## Abbreviations

The following abbreviations are used in this manuscript:

ABTS	2,2′-Azino-bis(3-ethylbenzothiazoline-6-sulfonic acid)
CCK-8	Cell Counting Kit-8
CDs	Carbon dots
CEPs	Circular economy principles
CFU	Colony forming unit
CO	Carbonyl group
COO^−^	Carboxylate group
CS	Chitosan
DNA	Deoxyribonucleic acid
DPPH	2,2-Diphenyl-1-picrylhydrazyl
EAB	Elongation at break
EFSA	European Food Safety Authority
EU	European Union
FDA	U.S. Food and Drug Administration
FRAP	Ferric reducing antioxidant power
FTIR	Fourier transform infrared spectroscopy
GL	Gelatin
GRAS	Generally Recognized As Safe
HDFa	Human dermal fibroblasts (adult)
HeLa	Henrietta Lacks (cervical cancer cell line)
HS27	Human skin fibroblast cell line (newborn foreskin)
MAP	Modified atmosphere packaging
MCA	Metal chelating activity
MBC	Minimum bactericidal concentration
MIC	Minimum inhibitory concentration
MTS	3-(4,5-dimethylthiazol-2-yl)-5-(3-carboxymethoxyphenyl)-2-(4-sulfophenyl)-2H-tetrazolium
MTT	3-(4,5-Dimethylthiazol-2-yl)-2,5-diphenyltetrazolium bromide
NMR	Nuclear magnetic resonance
OECD	Organisation for Economic Co-operation and Development
–OH	Hydroxyl group
PLE	Photoluminescence excitation
PUFA	Polyunsaturated fatty acids
ROS	Reactive oxygen species
RSA	Radical scavenging activity
SDG	Sustainable Development Goals
SEM	Scanning electron microscopy
SSO	Specific spoilage organisms
ST	Starch
TBARS	Thiobarbituric acid reactive substances
TEM	Transmission electron microscopy
TMA	Trimethylamine
UV	Ultraviolet
XRD	X-ray diffraction
